# Mathematical Simulation of Transport Kinetics of Tumor-Imaging Radiopharmaceutical ^99m^Tc-MIBI

**DOI:** 10.1155/2017/2414878

**Published:** 2017-06-15

**Authors:** O. N. Shevtsova, V. K. Shevtsova

**Affiliations:** ^1^Institute for Safety Problems of NPP, The National Academy of Sciences of Ukraine, Lisogirskaya Str. 12, Kyiv, Ukraine; ^2^National Medical University of O.O. Bogomolets, Pr. Peremogi 34, Kyiv, Ukraine

## Abstract

The proposed model describes in a quality way the process of tumor-imaging radiopharmaceutical ^99m^Tc-MIBI distribution with taking into account radiopharmaceutical accumulation, elimination, and radioactive decay. The dependencies of concentration versus the time are analyzed. The model can be easily tested by the concentration data of the radioactive pharmaceuticals in the blood measured at early time point and late time point of the scanning, and the obtained data can be used for determination of the washout rate coefficient which is one of the existing oncology diagnostics methods.

## 1. Introduction

The term “radiopharmaceutical” denotes the association of a radionuclide and pharmaceutical, that is symbiosis of biological, chemical, and physical properties.

Radiopharmaceuticals are approved for use in humans for diagnostic purposes chemical compounds whose molecules contain radionuclides. The method of diagnosis or radionuclide study of morphological and functional condition of the body using radionuclides or radionuclide-labeled indicators is one of the most common methods of detecting cancer. Radiopharmaceuticals are injected into the patient's body, and then using the radionuclide diagnostics devices it is possible to study the nature of moving, fixing, and removing them from the organs and tissues. Radiopharmaceuticals are selected with consideration of their radiopharmaceutical dynamic and nuclear-physical properties. Dynamics of radiopharmaceuticals is defined by a chemical compound that is the basis for radiopharmaceutical preparation. Registration of radiopharmaceutical is determined by the type of decay of the nuclide, by which it is marked. Some radiopharmaceuticals are called “radiotracers” because they are used only to diagnose (“trace”) dysfunctions in body tissues [1–4].

A radiopharmaceutical introduced into the body is firstly uniformly distributed in the blood [5] and then selectively trapped by certain organs and tissues.

The quantitative determination of blood volume can be performed by employing the tracer dilution method. ^125^I radioiodinated serum albumin (^125^I RISA), a radiopharmaceutical which disperses in the plasma, is used to determine plasma volume. ^51^Cr, a radiopharmaceutical that disperses within the cellular content of blood, is used to determine red cells volume (sometimes referred to as red cell mass) [5].

Radiopharmaceuticals which are selectively accumulated in tumor tissues are called the tumor-imaging agents. They are mainly included in cells with a high mitotic and metabolic activity. Due to high concentration of radiopharmaceuticals a tumor area will emerge on a scintigram, the graphic record obtained by scintigraphy, as a hot site. This research technique is called positive scintigraphy. Areas with increased accumulation of a radiotracer are called hot areas; usually they correspond to overactive functioning body areas, areas of hyperplasia, some types of tumors, and inflammatory tissue changes [6–11]. Radiopharmaceutical choice is caused by its pharmaceutical peculiarities and depends on tumor localization [6].

Radiopharmaceuticals are used in nuclear medicine as tracers for diagnostics and therapy of many diseases. Technetium 99m (^99m^Tc) serves as gamma-rays-emitting tracer nuclide for many radiopharmaceuticals. More than 30 different ^99m^Tc based radiopharmaceuticals are known, which are used for imaging and functional studies in various organs, for example brain, lung, kidneys, liver, and skeleton [7]. They also serve for diagnostic visualization of tumors. Their localization in the body is also determined by gamma-ray measurement.

### 1.1. Tumor-Imaging Agents Location

Tumor-imaging radiopharmaceuticals are characterized by some normal physiological fixation in tissues and organs; namely, ^99m^Tc-MIBI (often named as a tumor-imaging agent) is localized in myocardium (mainly, of the left ventricle of heart), kidney, and elements of the hepatobiliary system, spleen, and intestine. ^99m^Tc-MIBI and ^99m^Tc-tetrofosmin are accumulated in the primary tumor of the mammary gland. ^99m^Tc(V)DMSA is localized in kidneys, Schneiderian membrane, heart, and great vessels, caused by the blood pool; ^99m^Tc(V)-tectrotyd is localized in liver, kidneys, spleen, and thyroid gland [6].

### 1.2. Multidrug Resistance of Tumor Cells

Multidrug resistance of tumor cells is caused by ATP (adenosive-triphosphate) dependent decrease in cellular drug accumulation. ATP-transporters or ATP-binding cassette (ABC) transporters are the members of the largest transporter gene family* (ABCA-ABCG)* [12]. Their role is to use energy of ATP hydrolysis to translocate different substrates across biological membranes. One of the well-known ABC transporters is PGP (P-glycoprotein, gene symbol* ABCB1*), which is called a molecular “hydrophobic vacuum cleaner.” PGP is pulling substrates from the membrane and expelling them to promote multidrug resistance [13].

### 1.3. PGP and Its Physiological Role

PGP function is to force away hydrophobic drugs and peptides from the cell interior and protect tissues from toxic xenobiotics and endogenous metabolites at normal physiological conditions. PGP acts as the regulator of different structurally unbound substrates transport (e.g., toxins and anticancer agents) [14]. PGP takes part in the normal secretion of metabolites [15] and the transport function of PGP is necessary to protect cells from death. High PGP expression prevents stem-cell differentiation and extra expression of PGP delays apoptotic cascade [16]. So PGP may play essential role in regulation of apoptosis [16].

PGP is localized in normal human cells in various organs of human body and defines the direction of substrate transport [17, 18].

ABC transporters can be imaged in vivo with pharmaceuticals labeled with ^99m^Tc that are multidrug resistance substrates or inhibitors.

^99m^Tc-MIBI was the first radiopharmaceutical used as a substrate for ABC transporters to image the ABC expression in vivo [19].

### 1.4. Health or Disease Modulator:* ABCB1* Gene

*ABCB1* gene acts as a key player in processes of absorption, distribution, metabolism, excretion and drug-drug interaction. Therapeutic effectiveness can be affected by* ABCB1* gene variation that leads to overexpressing of PGP and to the result of increased efflux of anticancer drugs and to the drug resistance formation. Thus,* ABCB1* gene is characterized as a high polymorphic one and is considered as possible modulator of health or disease [14]. Variation of PGP expression depends on polymorphism of the human* ABCB1* gene. For people with the C/C genotype increased PGP expression is typical; possessors of the T/C genotype demonstrate slow intermediate PGP expression, and homozygous carriers of T/T genotype show restrained PGP [20, 21]. Researchers observed that, for individuals with T/T genotype which is associated with low PGP expression level, that is, the less efficiency to efflux the toxins, the high intracellular concentration of mutagens (a mutagen is a physical or chemical agent that changes the genetic materials, usually DNA, of an organism and thus increases the frequency of mutations above the natural background level) or toxins in the cell is realized that can lead to DNA damage and accumulation of mutations. Individuals with T/T genotype were found to be at increased risk of different kinds of oncological disease [14, 21–25]. Individuals with C/C genotype, associated with multidrug resistance, that is, activity of* ABCB1* forces cancer cells to become refractory with many agents that are PGP substrates, were found to have poor oncological disease prognosis [26–30].

## 2. Basics of In Vivo Dynamics of ^99m^Tc-MIBI and Methodological Details

### 2.1. Chemical Structure and Properties of ^99m^Tc-MIBI

Tc-99-sestamibi (hexakis-2-methoxy-isobutyl isonitrile, MIBI) or ^99m^Tc-MIBI was originally introduced for imaging myocardial perfusion. Chemical structure of ground state of ^99m^Tc-MIBI is a stable monovalent cation with a central Tc(I) core surrounded by 6 identical MIBI ligands, coordinated through the isonitrile carbons in an octahedral geometry. Alkyls isonitrile ligands encase the metal in a sphere of lipophilicity, while enabling delocalization of the cationic charge. This complex does not contain any ionisable functional groups and is extremely stable in vivo, without significant metabolism [28]. ^99m^Tc-MIBI is a substrate for PGP (P-glycoprotein, gene symbol* ABCB1*), MRP1 (multidrug resistance protein 1 (gene symbol* ABCC1*)), and MRP2 (multidrug resistance protein 2 (gene symbol* ABCC2*)) and can be used to image their expression in vivo [19]. Piwnica-Worms et al. [28] first demonstrated that ^99m^Tc-MIBI is a substrate for PGP and can be used as functional imaging agent for PGP in tumor xenografts in mice. Authors of [31] revealed that tumor retention of ^99m^Tc-MIBI is inversely proportional to the degree of PGP expression. ^99m^Tc-MIBI is effective for diagnosing breast cancer in patients with indeterminate mammography and dense breast [32], for imaging multidrug resistance in brain tumors, gastric cancer, head and neck cancer, and haematological malignancies [33].

### 2.2. Mechanism of ^99m^Tc-MIBI Accumulation and Retention

^99m^Tc-MIBI is a substrate for ATP-binding cassette (ABC) transporter proteins, including P-glycoprotein (PGP), and has been used to image their expression, mainly in cancer. Imaging PGP expression may have a role in renal transplantation and in investigation of the drug toxicity [24].

^99m^Tc-MIBI is a lipophilic cationic radiopharmaceutical that concentrates in the cells and inside mitochondria by active transport and passive diffusion. In metabolically hyperactive cells, the number of mitochondria is increased and, according to the level of cellular activity, different ^99m^Tc-MIBI can be observed [11, 28]. ^99m^Tc-MIBI is accumulated in tumor cells during the first minutes after intravenous injection [34–37].

^99m^Tc-MIBI is accumulated in cells with large numbers of mitochondria and in cytoplasm [34]. Mitochondria play an important role in energy metabolism and are integrally involved in embryonic development, cell signaling activities, cell-cycle control, and cell death [34]. ^99m^Tc-MIBI tissue uptake rate depends on tissue blood flow and cellularity. ^99m^Tc-MIBI kinetic is determined by apoptosis, proliferation, and angiogenesis; therefore ^99m^Tc-MIBI transport is used as an imaging biomarker of cellular metabolism in tumors [38, 39]. It should be noted that cancer cells have high antiapoptotic level that is caused by antiapoptotic protein Bcl-2 [39]. Tissue retention of ^99m^Tc-MIBI is a variable process because ^99m^Tc-MIBI is a substrate for PGP; as such it is determined obviously by tissue expression of PGP [28, 40].

Mechanism of tissue retention of ^99m^Tc-MIBI differs from ^99m^Tc-MIBI mechanism of washout or elimination. Elimination of ^99m^Tc-MIBI from cells is determined by the activity of drug transporters.

### 2.3. Physical Processes of ^99m^Tc-MIBI Penetration and Accumulation

^99m^Tc-MIBI is a lipid soluble small organic cation. The electrical membrane potential causes ^99m^Tc-MIBI accumulation mechanism [36], determined by sarcolemmic and mitochondrial potentials. Since cell membranes are more negatively charged at the mitochondrial level than potential at sarcolemma level (sarcolemma is the plasma membranes of muscle fibers), the radiopharmaceuticals are mainly localized in mitochondria.

**Figure 1:**
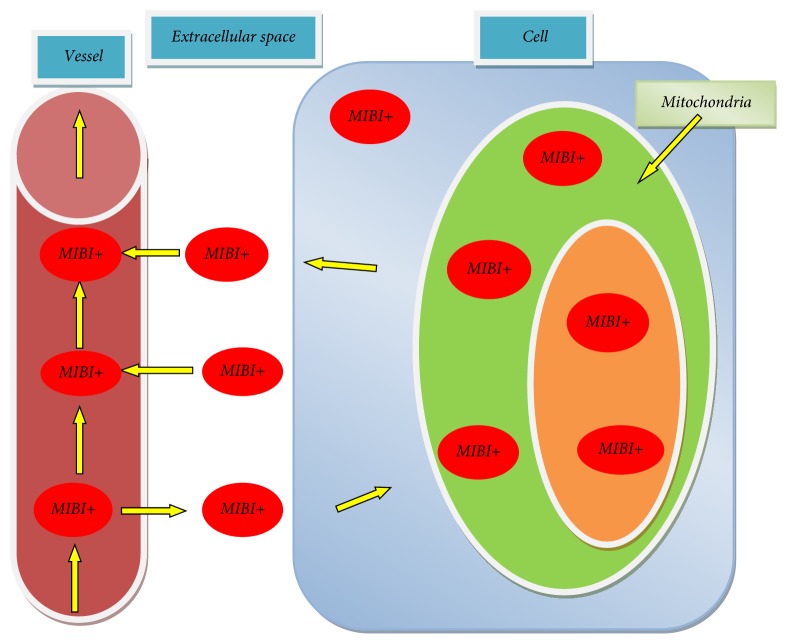
^99m^Tc-MIBI transport kinetic in a cell.

The mechanism of ^99m^Tc-MIBI accumulation in the myocardium can be explained by intracellular mitochondria electrophilic capture of radiopharmaceuticals. ^99m^Tc-MIBI penetration from the blood through the cell membrane into the cell has a diffuse character (diffusion is directed along a concentration gradient), and then ^99m^Tc-MIBI is captured by the active mitochondria. ^99m^Tc-MIBI transport kinetic is illustrated in Figure 1 [36].

Permeability of the endothelium of the blood vessels for ^99m^Tc-MIBI is high [41, 42]. The combination of these mechanisms ensures high capture and lasting intracellular retention of radiopharmaceuticals and fragments of its metabolism in tumor tissue. Due to practically selective ^99m^Tc-MIBI localization, ^99m^Tc-MIBI accumulation level is a characteristic of the tissue metabolic activity.

Accumulation mechanism of ^99m^Tc-MIBI is similar to the cytostatic mechanism of accumulation [28, 43, 44]. P-Glycoprotein which is present in tumor cells and which helps cytostatic elimination from the cells uses ^99m^Tc-MIBI as the substrate.

### 2.4. ^99m^Tc-MIBI Washout Rate Coefficient

^99m^Tc-MIBI is characterized by typical high capture at the first passage of tumor bloodstream, and the blood flow is the limiting factor for the amount of radiopharmaceuticals that are accumulated in the tissues.

Preoperative washout rates of the ^99m^Tc-MIBI (investigation of the primary breast tumor) correlated with levels of PGP detected in the surgically resected specimens. According to scintigraphic data for patients with breast cancer overexpressing PGP the ^99m^Tc-MIBI washout rate coefficient is threefold higher than for patients not expressing PGP breast cancer [45, 46].

In clinical practice ^99m^Tc-MIBI transport kinetic is estimated by the washout rate coefficient *K*_WOR_ [47].

(1) $$\begin{array}{c} K_{WOR}=\frac{\left(A_{1}-F_{1}\right)-\left(A_{2}-F_{2}\right)}{\left(A_{1}-F_{1}\right)}=1-\frac{A_{2}-F_{2}}{A_{1}-F_{1}}, \end{array}$$

where *A*_1_,  *F*_1_ are the ^99m^Tc-MIBI MIBI pharmaceutical concentrations in tumor and normal cells; correspondingly, in the first minutes of the radiopharmaceutical introduction, *A*_2_,  *F*_2_ are the ^99m^Tc-MIBI radiopharmaceutical concentrations in tumor and normal cells after 60 ÷ 240 minutes. If *K*_WOR_ < 0.45 the PGP concentration is low in tumor cells; if *K*_WOR_ > 0.45 the PGP concentration in tumor cells is high [47, 48].

### 2.5. Medical Information Obtained by the Radiotracer Method

Radiotracer simulation is one of the main methods of interpretation of radionuclide research results. Quantitative data of radiotracer transport kinetics in the body are presented in the form of “activity-time” or “concentration-time” which reflect the spatial and temporal processes of change in the concentration of radioactive indicator in the “regions of interest” and characterize the rate of ^99m^Tc-MIBI retention and washout in the organ or tissue. This makes it possible to monitor changes of scintigraphy images as a function of time to assess relevant indicators of the various functions of organs and tissues which are under the treatment. The complexity of this mathematical simulation, on one hand, is the excessive simplification of the anatomical features of the organism when it is divided into kinetic chambers, which can lead to loss or distortion of information important for diagnostics. On the other hand, excessive consideration of all possible interrelationships in the functioning of organs and systems results in excessive number of mathematical data useless for the clinical interpretation.

The “time-activity” curves of radiotracer transport kinetic can be conditionally divided into three segments [49]:

- Vascular segment corresponds to the rapid growth of the observed curve in the first seconds after injection of a radiotracer, which reflects the intake of the radiotracers in vascular and beginning the process of radiotracer accumulation. Vascular segment depends on the blood flow.
- Secretory segment corresponds to the smooth amplitude growing up to the maximum value and then entering the plateau phase, which reflects the processes of the radiotracer accumulation and retention (the plateau part of the curve).
- Excretory segment of the curve corresponds to the declining part of the curve, reflecting the process of radiotracer washout.

After intravenous injection radiotracers are distributed and accumulated in the body; that is, they create free and bounded fractions of radiotracers, and both of them are located in the “region of interest.”

Complex radionuclide investigation includes nondirect angiography, slow dynamic scintigraphy, and scanning of the whole body [49]. The “activity-time” curves obtained during dynamical scintigraphy of normal tissue and dynamic curves obtained in metastatic areas are characterized by different intensities. Subtraction of the “time-activity” curves of the tissue background from the “activity-time” curves obtained from metastases areas allows us to evaluate real accumulation of radiotracers in tumor cells [50].

^99m^Tc-MIBI radiotracer is distributed, accumulated/retained, and eliminated in normal and tumor tissues in different ways. To evaluate real situation in an organ damaged by tumor it is necessary to locate the tumor region or “the region of interest.” For this purpose we propose a simple mathematical model that describes the ^99m^Tc-MIBI radiotracer transport kinetics taking into account radioactive decay of the radiotracer, and different types of the radiotracer transport kinetics in normal and tumor cells.

### 2.6. Mathematical Simulation of Radiotracer Transport Kinetics

Radiotracer kinetic simulation was studied by many authors [51–54].

In a typical PET study, PET data are sequentially obtained after the radioactive tracer is introduced (usually administrated intravenously) over time. The interpretation of the observed PET data over time is fulfilled in the frame of the “compartments model,” where “compartments” mean physiologically separate pools of a tracer substance. Authors [51] represented general four compartments' model or three tissue compartments' model. The first compartment is the arterial blood. From arterial blood, the radioligand passes into the second compartment, known as the free compartment. The third compartment is the region of specific binding which we are usually interested to observe. The fourth compartment is a nonspecific-binding compartment that exchanges with the free compartment. The transport and binding rates of the tracer are assumed to be linearly related to the concentration differences between two compartments. Data obtained by PET detectors are obtained as the sum of these compartments. The parameters can be estimated by fitting the model to measured PET data with arterial radioactivity concentration as the input function. However this method requires the frequent manual sampling of the arterial blood or continuous radioactivity monitoring by external radiation detectors.

Compartmental model can be calculated by Laplace Transform method [52]. Compartment model can be reversible or irreversible (containing at least one compartment which has no outlet).

Application of radiotracer kinetic simulation allows us to substantiate quantitative results obtained during radionuclide diagnostic and to connect them with morphological and functional characteristics of liver and bile-excreting systems and haemodynamics indicators [53].

Duration of a radiotracer exposure depends on the radiotracer decay rate *λ* if the compartment is closed (without elimination of radiotracer) or the radiotracer decay rate *λ* and biological elimination rate if the compartment is opened. In the second case the general elimination rate is higher. After intravenous bolus injection the radiotracer moves from one compartment to the next one. Radiotracer kinetics is described by the system of differential equations [53, 54]. The solution of such system is determination of the effective rate of accumulation/elimination.

The next stage is to find all the points and use the approximation method (e.g., the smallest square). Standard approach is to apply the functional of the residual function. For the accumulation function Lunev [54] has proposed the following functional Φ = ∑_*i*_(*F*(*t*_*i*_) − *F*(*t*_*i*_^*e*^)), lim_*t*→*∞*_⁡Φ → min, where *F*(*t*_*i*_) is the function describing the real accumulation/elimination of the radiotracer, *F*(*t*_*i*_^*e*^) is the approximation exponential function, and the limit describes the minimization of the sum of squared deviations to the curve. The model is rather complicated. The aim of the calculation is to determine the area under the curve, the physical sense of which is the number of decays in each compartment. The elimination rate of a radiotracer is described by the ratio of the introduced activity to the value of the area under the curve or clearance. It is obvious that the higher clearance means the lower exposure of an organ.

### 2.7. Simulation of Radiopharmaceutical Transport Kinetics in the Body

After bolus intravenous injection of the radiotracers in the body the process of transferring the radiotracers by blood vessels is begun, and the so-called radiotracer “dilution” process is realized, namely, the absorption of radiotracers by other organs and tissues and radiotracers decay. (Bolus is a certain amount of medicine, injected into the body intravenously. The injected bolus quite quickly causes a response reaction in the body.)

The part of radiopharmaceuticals which is absorbed by cells is immediately metabolized, and metabolic products quickly returned to the general blood circulation. The processes taken into account in this model are as follows:

(1) radioactive decay of pharmaceuticals; (2) accumulation of pharmaceuticals in the tumor cells; (3) accumulation of pharmaceuticals in the normal cells; (4) transport of pharmaceuticals from the blood vessels; (5) transport of pharmaceuticals and metabolites from the normal cells in the blood vessels; (6) transport of pharmaceuticals and metabolites from the tumor cells in the blood vessels.

The model of transport kinetics of radiotracers is described by a system of differential equations for radiopharmaceutical concentration levels in blood vessels, in tumor cells, and in normal cells (background activity) [49]. The system of equations describes the processes of accumulation/retention of a radiotracer in the cells, the radiotracer elimination/washout, and radiotracer radioactive decay. Elimination is described by kinetic equations of the 1st order: the content of drug in the blood is proportional to its concentration:

(2) dxdt=−λdydt=−λdzdt=−λ

where *λ* is the radioactive decay constant of radiopharmaceuticals, *E* is the elimination constant of radiotracers, *x*(*t*) is the concentration of radiotracers in tumor cells, *y*(*t*) is the concentration of radiotracers in normal cells (the background concentration), *z*(*t*) is the concentration of radiotracers in blood vessels, *β*_*zx*_ is the rate of radiotracer capture by tumor cells, or an effective rate of blood flow which depends on the degree of branching of blood vessels, *β*_*zy*_ is the rate of the radiotracer capture by normal cells, *β*_*xz*_ is the elimination rate of radiotracers from tumor cells in the bloodstream, that is, the reciprocal resistance of tumor cells, and *β*_*yz*_ is the elimination rate of radiotracers from normal cells in the bloodstream, that is, the reciprocal resistance of normal cells.

The study fulfilled in the paper covers the radiopharmaceutical transport kinetics only in the case when chemical resistance of tumor cells is higher than chemical resistance of normal cells, 1/*β*_*xz*_ > 1/*β*_*yz*_, or in the terms of the reciprocal chemical resistance *β*_*xz*_ < *β*_*yz*_ [55]. According to [55] the ratio of the capture coefficient in the tumor and normal cells is located in the range of 3 ÷ 4. Optimal visualization of tumor neoplasms by radiopharmaceuticals is fulfilled at the condition when accumulation of radiopharmaceuticals by normal cells is low (*β*_*zy*_ < *β*_*zx*_) [55]. The simulation parameters were chosen with taking into account this factor, *β*_*zy*_ = (1/2)*β*_*zx*_. Half-decay period of ^99m^Tc-MIBI is equal to *T*_1/2_ = 6 hours.

## 3. Results and Discussions

### 3.1. Model Verification

We consider in this model the initial situation when all radiopharmaceuticals are located in a blood vessel (the initial conditions: *x*(0) = 0,  *y*(0) = 0,  *z*(0) = *n*_0_, where *n*_0_ is the initial radiopharmaceutical amount). The simulation results will be presented in a form of the “concentration-time” curves. This paper covers some set of model situations which can be realized in the frame of the assumptions accepted in the model. Description of the processes and explanation for each figure are presented under the figure.

As the first step of the model verification we will test its correctness for qualitative description of transport kinetics of intravenously administrated arbitrary pharmaceuticals.

### 3.2. Nonradioactive Pharmaceuticals Transport Kinetics without Washout

Processes which can be realized in this case are accumulation of pharmaceuticals in the cells of different nature (tumor and normal), without further elimination. The key parameters are *β*_*zy*_ = (1/2)*β*_*zx*_ = 1/2,  *E* = 0,  *λ* = 0.

**Figure 2:**
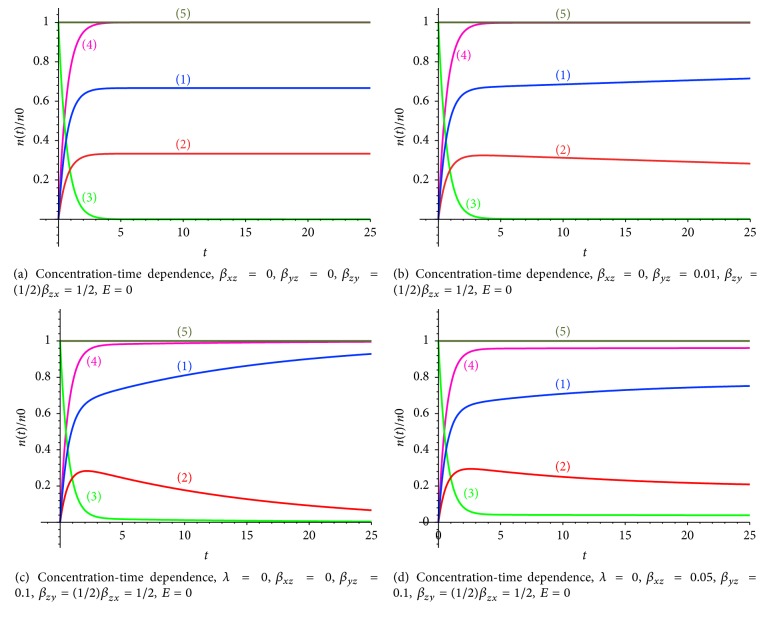
(1) The pharmaceutical concentration in tumor cells *n*_*x*_, (2) the pharmaceutical concentration in normal cells *n*_*y*_, (3) the pharmaceutical concentration in blood vessels *n*_*z*_, (4) the total pharmaceutical concentration in tumor and normal cells *n*_*x*_ + *n*_*y*_, and (5) the total pharmaceutical concentration in tumor and normal cells and in the blood vessels *n*_*x*_ + *n*_*y*_ + *n*_*z*_.

If the pharmaceutical elimination from tumor and normal cells is absent (*β*_*xz*_ = 0,  *β*_*yz*_ = 0), then the whole amount of the pharmaceuticals is accumulated in the body; that is, the pharmaceutical mass conservation law is fulfilled: *x*(*t*) + *y*(*t*) + *z*(*t*) = *n*(*t*) = *n*_0_. The results of simulation of pharmaceutical concentration dynamics are presented in Figures 2(a)–2(d).

Pharmaceuticals from the blood stream are retained by tumor and normal cells in the case of absence of elimination, so the pharmaceutical accumulation process in tumor (Figure 2(a), curve (1)) and normal cells (Figure 2(a), curve (2)) is realized. It should be noted that only a part of the pharmaceuticals can be captured and retained by a cell. High enough concentration of the substrate (^99m^Tc-MIBI) can lead to initiation of the competition process between incoming substrate molecules and reaction-response of the cell on the external chemical irritant. So the coefficient of accumulation can be the function of the accumulated pharmaceuticals, and the reason is caused by the limitation for accumulation of a pharmaceutical amount in a cell.

Let us consider the case of low resistivity of normal cells and high resistivity of tumor cells. In this situation the part of radiopharmaceuticals (the free fraction) localized near normal cells returns back into the blood vessels, and then this part starts to be captured by the tumor cells that leads to the increase of the pharmaceutical concentration in tumor cells and the decrease of the pharmaceutical concentration in normal cells at absence of elimination (Figures 2(b) and 2(c)). If *β*_*yz*_ (Figure 2(c)) is increased in comparison with *β*_*yz*_ (Figure 2(b)), the part of pharmaceuticals captured by tumor cells is higher, and correspondingly, the part of the pharmaceuticals captured by normal cells is lower.

If a part of radiopharmaceuticals localized near tumor and normal cells returns back into the blood vessels, then this part is transferred by the blood and pharmaceuticals start to be captured again by tumor and normal cells; that is, the secondary redistribution of pharmaceuticals is realized (Figures 2(c) and 2(d)), and we have other values of the pharmaceutical concentration in tumor and normal cells.

### 3.3. Nonradioactive Pharmaceutical Transport Kinetics with Elimination

**Figure 3:**
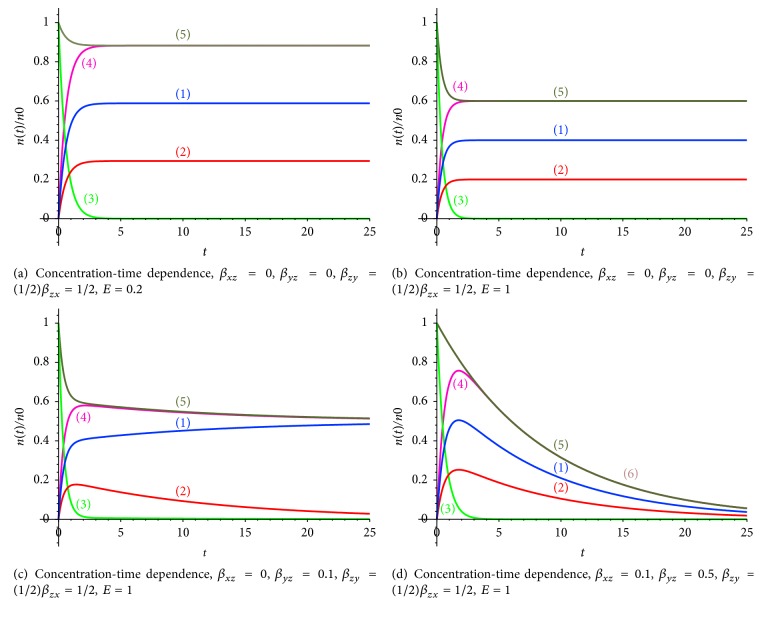
(1) The pharmaceutical concentration in tumor cells *n*_*x*_, (2) the pharmaceutical concentration in normal cells *n*_*y*_, (3) the pharmaceutical concentration in blood vessels *n*_*z*_, (4) the total pharmaceutical concentration in tumor and normal cells *n*_*x*_ + *n*_*y*_, and (5) the total pharmaceutical concentration in tumor and normal cells and in the blood vessels *n*_*x*_ + *n*_*y*_ + *n*_*z*_.

Processes of the secondary capture of pharmaceuticals by normal and tumor cells with taking into account the elimination process and the different resistance of tumor and normal cells are illustrated in Figures 3(a)–3(d). If the tumor cells resistance is high enough (*β*_*xz*_ = 0), then the escape of pharmaceuticals is caused by the elimination only (Figures 3(a)–3(c)). The decrease of the tumor cells resistance (*β*_*xz*_ = 0,1) leads to participation of two mechanisms: the elimination and the escape of pharmaceuticals from the cells and the secondary capture effect (Figure 3(d)).

### 3.4. ^99m^Tc-MIBI Transport Kinetics Model

We will consider in this model the case of bolus intravenous injection when radiopharmaceuticals are located in the bloodstream (the initial conditions: *x*(0) = 0,  *y*(0) = 0,  *z*(0) = *n*_0_, where *n*_0_ is the initial radiopharmaceutical amount. Half-decay period of ^99m^Tc-MIBI is equal to *T*_1/2_ = 6 hours.

#### 3.4.1. ^99m^Tc-MIBI Transport Kinetic Model in Absence of Radiopharmaceutical Elimination

**Figure 4:**
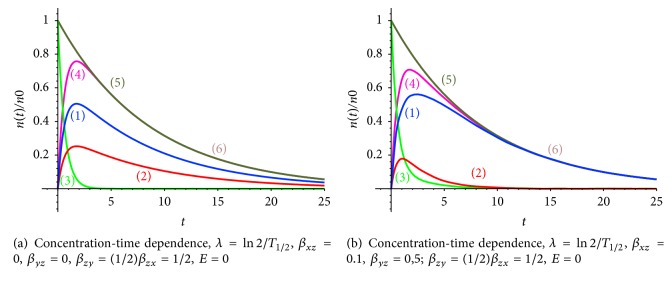
(1) Radiopharmaceuticals (radiotracers) concentration in tumor cells *n*_*x*_, (2) radiotracer concentration in normal cells *n*_*y*_, (3) radiotracer concentration in the blood *n*_*z*_, (4) total concentration of radiotracer in the tumor and normal cells *n*_*x*_ + *n*_*y*_, (5) the total concentration of radiotracer in the tumor and normal cells and blood vessels *n*_*x*_ + *n*_*y*_ + *n*_*z*_, and (6) the concentration of radiopharmaceuticals *n* = *n*_0_*e*^−(ln⁡2/*T*_1/2_)*t*^.

**Figure 5:**
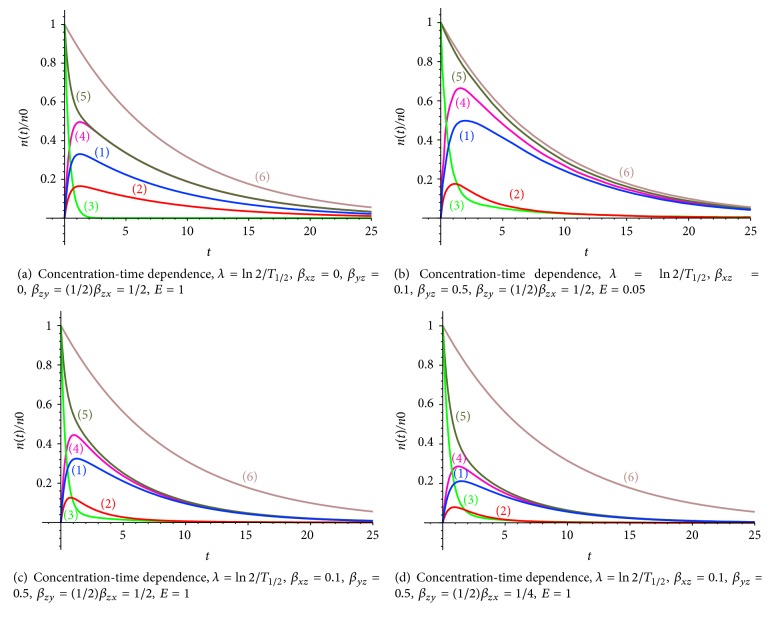
(1) Radiopharmaceuticals (radiotracers) concentration in tumor cells *n*_*x*_, (2) radiotracer concentration in normal cells *n*_*y*_, (3) radiotracer concentration in the blood *n*_*z*_, (4) total concentration of radiotracer in the tumor and normal cells *n*_*x*_ + *n*_*y*_, (5) the total concentration of radiotracer in the tumor and normal cells and blood vessels *n*_*x*_ + *n*_*y*_ + *n*_*z*_, and (6) the concentration of radiopharmaceuticals *n* = *n*_0_*e*^−(ln⁡2/*T*_1/2_)*t*^.

**Figure 6:**
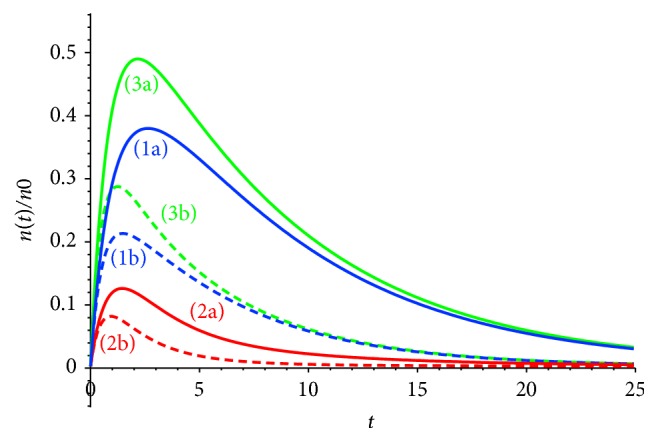
(1) Radiopharmaceuticals (radiotracers) concentration in tumor cells *n*_*x*_, (2) radiotracer concentration in normal cells *n*_*y*_, (3) total concentration of radiotracers in the tumor and normal cells *n*_*x*_ + *n*_*y*_. Concentration-time dependence, (a) *k*_WOR_ = 0.5,  *λ* = ln⁡2/*T*_1/2_,  *β*_*xz*_ = 0.1,  *β*_*yz*_ = 0.5,  *β*_*zy*_ = (1/2)*β*_*zx*_ = 1/4,  *E* = 0.1. (b) *k*_WOR_ = 0.7,  *λ* = ln⁡2/*T*_1/2_,  *β*_*xz*_ = 0.1,  *β*_*yz*_ = 0.5,  *β*_*zy*_ = (1/2)*β*_*zx*_ = 1/4,  *E* = 1.

**Figure 7:**
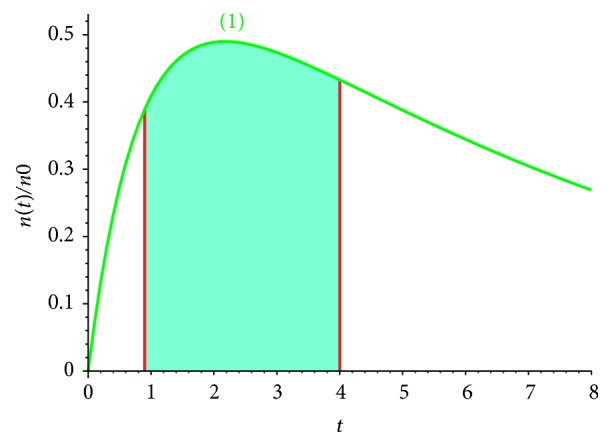
(1) Total concentration of radiotracers in the tumor and normal cells *n*_*x*_ + *n*_*y*_. Concentration-time dependence, *k*_WOR_ = 0.5,  *λ* = ln⁡2/*T*_1/2_,  *β*_*xz*_ = 0.1,  *β*_*yz*_ = 0.5, *β*_*zy*_ = (1/2)*β*_*zx*_ = 1/4,  *E* = 0.1.

If radiopharmaceutical elimination from tumor and normal cells is absent (*β*_*xz*_ = 0,  *β*_*yz*_ = 0), the pharmaceutical is accumulated in the body, and the mass conservation law of the pharmaceutical amount is fulfilled: *x*(*t*) + *y*(*t*) + *z*(*t*) = *n* = *n*_0_*e*^−*λt*^ = *n*_0_*e*^−*t*ln⁡2/*T*_1/2_^. Putting together all the equations of the system (2), one can obtain the equation of the radioactive decay of the radiopharmaceuticals: *dx*/*dt* + *dy*/*dt* + *dz*/*dt* = −*λ*(*x*(*t*) + *y*(*t*) + *z*(*t*)) = −*λn*(*t*). The simulation results of the radiopharmaceuticals concentration versus time (in hours) are presented in Figures 4(a)-4(b), 5(a)–5(d), 6, and 7. Curves (5) and (6) (Figures 4(a)-4(b)) coincide if the radiopharmaceutical elimination is absent. The concentration of radiopharmaceuticals in tumor and normal cells with taking into account the process of escape of radiopharmaceuticals from the tumor and normal cells in the blood vessels and subsequent redistribution of radiopharmaceuticals is taken into account (Figure 4(b)).

#### 3.4.2. Radiopharmaceutical Transport Kinetic Taking into Account Radiopharmaceutical Elimination

The character of radiopharmaceuticals distribution is as follows: radiopharmaceuticals quickly leave the bloodstream (Figures 5(a)–5(d)) (curve (3)) and are accumulated in the tumor cells (curve (1)) and normal cells (curve (2)).

Let us consider 2 possible situations: (1) Radiopharmaceuticals are accumulated in the tumor and normal cells and the radioactive decay and the elimination processes are realized (Figure 5(a)). (2) Radiopharmaceuticals are accumulated in the tumor and normal cells and the radioactive decay and the elimination processes are realized and the process of escape of radiopharmaceuticals from the tumor and normal cells in the blood vessels and subsequent redistribution of radiopharmaceuticals is taken into account (Figure 5(b)).

From comparison of Figures 5(b) and 5(c) (different elimination ratio coefficients *E*) it follows that the elimination ratio of the pharmaceuticals determinates the amplitude of the curves (1, 3, 4) (other parameters are the same; redistribution process is taken into account as well). However, if the ratio of the rate of the radiotracer capture by normal cells *β*_*zy*_ to the rate of the radiotracer capture by tumor cells *β*_*xz*_ (*β*_*zy*_ = (1/2)*β*_*zx*_ = 1/4) is decreased, the amplitude of the curves (1, 2, 4) became small enough. The behavior of the curves means that tumor cells capture smaller amount of radiopharmaceuticals, which leave the body by radioactive decay and elimination (Figures 5(b)-5(c)); that is, at a higher rate of radiopharmaceuticals escape from tumor and normal cells, qualitative behavior of the curves “concentration-time” remains the same, but the amplitudes of curves (1) and (2) are decreased.

Let us analyze radiopharmaceutical accumulation versus the rate of radiopharmaceutical capture by tumor and normal cells *β*_*zx*_,  *β*_*zy*_ (Figures 5(c)-5(d)). The rate of radiopharmaceutical capture determines the amplitude of a curve; the increase of *E* leads to the decrease of the height and width of the plateau (Figure 6, curves (1a) and (1b), and curves (2a) and (2b)).

Comparison of situations (a) and (b) (Figure 6) shows that the increase of the washout coefficients leads to the decrease of the accumulation level and to early appearance of the plateau at the curve. The width of the plateau is also decreased.

The summary concentration-time dependence (Figure 7, curve (1)) illustrates the start and the end points of the diagnostic, and the area under the curve determinates the clearance value that depends on the elimination coefficient (Figure 6). In this model it is easy to estimate the washout rate coefficient *K*_WOR_ as the area under the curve (the total concentration of radiopharmaceutical in the tumor and normal cells), limited by vertical asymptotes, corresponding to the time point, when maximum value of the radiopharmaceutical concentration is reached, and the second time point, the point of termination of the diagnostic process, the first of these points corresponds to a maximum accumulation of radiopharmaceutical, and the second one describes the final time of the diagnostic study. The washout rate coefficient *K*_WOR_ was estimated in the presented model.

## 4. Conclusions

The proposed model correctly describes in a qualitative way the process of tumor-imaging radiopharmaceutical ^99m^Tc-MIBI distribution with taking into account not only radiopharmaceutical accumulation and elimination, but also radioactive decay. The dependencies of activity versus time are analyzed. The model can be easily tested by the concentration data of the radioactive pharmaceuticals in the blood measured at early time point and late time point of the scanning, and the obtained data can be used for determination of the washout rate coefficient which is one of the existing oncology diagnostics methods.

Integration of the mathematical model with experimental or clinical data can provide better tool to understand the radiopharmaceuticals distribution, accumulation, and elimination processes, in particular, to evaluate time of accumulation and retention of ^99m^Tc-MIBI in the “region of interest.” The use of radiotracer transport kinetic models will make it possible to connect some physical indicators with definite physiological processes to evaluate the investigation results and present the objective evaluation of functional state of the investigated organ according to the obtained radiological data.
